# Frontostriatal Dysfunction During Decision Making in Attention-Deficit/Hyperactivity Disorder and Obsessive-Compulsive Disorder

**DOI:** 10.1016/j.bpsc.2018.03.009

**Published:** 2018-08

**Authors:** Luke J. Norman, Christina O. Carlisi, Anastasia Christakou, Clodagh M. Murphy, Kaylita Chantiluke, Vincent Giampietro, Andrew Simmons, Michael Brammer, David Mataix-Cols, Katya Rubia

**Affiliations:** a Department of Child and Adolescent Psychiatry, Institute of Psychiatry, Psychology and Neuroscience, King's College, London, United Kingdom; b Department of Forensic and Neurodevelopmental Sciences, Sackler Institute for Translational Neurodevelopmental Sciences, Institute of Psychiatry, Psychology and Neuroscience, King's College, London, United Kingdom; c Department of Neuroimaging, Institute of Psychiatry, Psychology and Neuroscience, King's College, London, United Kingdom; d National Institute for Health Research Biomedical Research Centre for Mental Health, South London and Maudsley NHS Foundation Trust and Institute of Psychiatry, Psychology and Neuroscience, King's College London, London, United Kingdom; e Division of Psychology and Language Sciences, Department of Clinical, Education and Health Psychology, University College London, London, United Kingdom; f Behavioural Genetics Clinic, Adult Autism Service, Behavioural and Developmental Psychiatry Clinical Academic Group, South London and Maudsley Foundation NHS Trust, London, United Kingdom; g Centre for Integrative Neuroscience and Neurodynamics, School of Psychology and Clinical Language Sciences, University of Reading, Reading, United Kingdom; h Department of Psychiatry, University of Michigan, Ann Arbor, Michigan; i Department of Neurobiology, Care Sciences and Society, Center for Alzheimer Research, Division of Clinical Geriatrics, Karolinska Institutet, Stockholm, Sweden; j Department of Clinical Neuroscience, Centre for Psychiatry Research, Karolinska Institutet, Stockholm, Sweden

**Keywords:** ADHD, Computational modeling, Disorder specificity, fMRI, OCD, Reward

## Abstract

**Background:**

The aim of the current paper is to provide the first comparison of computational mechanisms and neurofunctional substrates in adolescents with attention-deficit/hyperactivity disorder (ADHD) and adolescents with obsessive-compulsive disorder (OCD) during decision making under ambiguity.

**Methods:**

Sixteen boys with ADHD, 20 boys with OCD, and 20 matched control subjects (12–18 years of age) completed a functional magnetic resonance imaging version of the Iowa Gambling Task. Brain activation was compared between groups using three-way analysis of covariance. Hierarchical Bayesian analysis was used to compare computational modeling parameters between groups.

**Results:**

Patient groups shared reduced choice consistency and relied less on reinforcement learning during decision making relative to control subjects, while adolescents with ADHD alone demonstrated increased reward sensitivity. During advantageous choices, both disorders shared underactivation in ventral striatum, while OCD patients showed disorder-specific underactivation in the ventromedial orbitofrontal cortex. During outcome evaluation, shared underactivation to losses in patients relative to control subjects was found in the medial prefrontal cortex and shared underactivation to wins was found in the left putamen/caudate. ADHD boys showed disorder-specific dysfunction in the right putamen/caudate, which was activated more to losses in patients with ADHD but more to wins in control subjects.

**Conclusions:**

The findings suggest shared deficits in using learned reward expectancies to guide decision making, as well as shared dysfunction in medio-fronto-striato-limbic brain regions. However, findings of unique dysfunction in the ventromedial orbitofrontal cortex in OCD and in the right putamen in ADHD indicate additional, disorder-specific abnormalities and extend similar findings from inhibitory control tasks in the disorders to the domain of decision making under ambiguity.

Attention-deficit/hyperactivity disorder (ADHD) is defined by age-inappropriate problems with inattention, impulsivity, and hyperactivity (1) and affects 3% to 8% of children worldwide as well as 4% of adults (2). Obsessive-compulsive disorder (OCD), on the other hand, is characterized by obsessions, defined as recurrent and intrusive thoughts (e.g., on themes of contamination, checking, orderliness, and symmetry), and compulsions, i.e., repetitive, ego-dystonic, and time-consuming behavioral and mental rituals (e.g., repetitive washing or checking) (1). OCD has a lifetime prevalence of 2% to 3% (3).

In early models, ADHD and OCD were placed at opposing ends of a hypothesized impulsivity-compulsivity spectrum 4, 5. However, the high level of comorbidity between the disorders in particular during adolescence does not fit with this model and suggests a potential overlap in the two disorders in genetic and neuroendophenotypic features 3, 4, 5, 6, 7. For instance, both ADHD and OCD patients show neural dysfunction during decision making and reward processing 8, 9, 10, 11, 12, 13, 14, with this proposed to underlie impulsive behaviors in ADHD and compulsive behaviors in OCD 15, 16, 17. Recent efforts including the Research Domain Criteria emphasize the importance of investigating transdiagnostic phenotypes that may be underpinned by shared and/or disorder-specific neurofunctional mechanisms (18).

One of the most commonly used tasks to measure reward-based decision making is the Iowa Gambling Task (IGT), in which participants are instructed to select cards one at a time from one of four possible decks 19, 20. Each card is associated with a monetary win or loss, and participants must learn to choose from advantageous decks and avoid disadvantageous decks (21). Computational models of IGT performance suggest that a number of cognitive functions underlie individual differences in task performance, including the ability to learn and utilize the reinforcement contingencies of each deck, reinforcement learning rates, reward and loss sensitivity, relative use of a win-stay/lose-shift–based versus expectancy-based choice selection strategy, and a tendency to exploit advantageous decks versus continued exploration of alternative options 22, 23.

In the brain, performance during decision making on the IGT recruits activation in the ventromedial orbitofrontal cortex (vmOFC) and the ventral striatum (VS), regions that are closely interconnected as part of a dopaminergic mesolimbic circuit 21, 24, 25, 26. In tandem, these brain regions support flexible emotional learning and guide decision making by encoding prospective values for available options 21, 24, 27, 28. Performance on the IGT also requires assessment of rewards and losses, which recruits the vmOFC, VS, and adjacent limbic regions 21, 24, 29, 30. During adolescence, performance on decision-making tasks such as the IGT improves in a linear fashion, independently from maturations in dorsolateral prefrontal cortex (PFC)–dependent executive functioning, and in line with maturation of the vmOFC and VS 24, 31, 32, 33, 34.

The aim of this study was to conduct a comparison of neurofunctional abnormalities during performance of the IGT in adolescent ADHD and OCD patients. In both patient groups we anticipated an impaired ability to align deck choice with expected values 13, 35, as well as altered striatal activation during decision making 9, 36, 37. Given the previous literature on orbitofrontal dysfunction in OCD, it was predicted to be more pronounced in or disorder-specific to adolescents with OCD in the current study 7, 38, 39, 40. During outcome processing, decreased VS responses to rewards were anticipated in adolescents with OCD, while ADHD adolescents were expected to show increased VS responses to rewards 8, 41, 42, 43, 44.

## Methods and Materials

### Participants

Fifty-six (16 ADHD, 20 OCD, 20 control subjects) right-handed (45) male adolescents aged between 12 and 18 years of age participated, with an IQ >80 as measured by the Wechsler Abbreviated Scale of Intelligence-Revised short form (46). ADHD boys met DSM-IV criteria for inattentive/hyperactive-impulsive combined subtype, as assessed using the standardized Maudsley diagnostic interview 1, 47, scored above clinical cutoff on the Conners’ Parent Rating Scale-Revised (48) as well as the inattention/hyperactivity scale of the Strengths and Difficulties Questionnaire (49), and were recruited from local Child and Adolescent Mental Health Services. Medicated ADHD patients underwent a 48-hour washout period before scanning. Patients with ADHD were free of comorbidities besides conduct disorder, as determined by a consultant psychiatrist. Boys with OCD were recruited from a national specialist clinic for childhood OCD and local Child and Adolescent Mental Health Services and had clinical diagnoses of OCD, as assessed according to the ICD-10 criteria and the Children’s Yale-Brown Obsessive Compulsive Scale (CY-BOCS) (49). Following a detailed clinical assessment, consisting of in-depth interviews with both patient and parents, patients with OCD were determined by a consultant psychiatrist to be free of comorbid diagnoses, including comorbid ADHD.

Control participants had no diagnoses of any psychiatric conditions and were recruited using local advertising. Data for some participants have been published elsewhere 21, 24, 35.

The study was conducted in accordance with the Declaration of Helsinki. Ethical approval was obtained from the local Research Ethics Committee (05/Q0706/275). Study details were explained to both child and guardian, and written informed consent was obtained for all participants.

### IGT Paradigm

Participants were presented with four decks of cards (labeled A, B, C, and D) on a computer screen and asked to select one of the decks by pressing with their right hand one of four buttons. Participants completed 80 trials and were instructed to win as much money as possible and lose as little money as possible. Participants were not informed of how many trials they would perform. There was a 50% probability of winning on each deck. Decks A and B (disadvantageous decks) gave relatively large gains (£190, £200, or £210) but even larger losses (£240, £250, or £260), whereas decks C and D (advantageous decks) gave small gains (£90, £100, or £110) but even smaller losses (£40, £50, or £60). A £2,000 “loan” and running total were presented at the bottom of the task display.

Each trial of the IGT is divided as follows: 1) the choice phase, 2) a 6-second delay between choosing a deck and being presented with the outcome, and 3) the 3-second outcome evaluation phase. Total trial length was 15 seconds, ending with a blank screen after outcome presentation that served as an implicit baseline in the functional magnetic resonance imaging (fMRI) analysis (Supplemental Figure S1).

Participants were informed that performance on the task determined the amount of money they would receive at the end of the session. In fact, all participants received the full amount (£30). Participants were acclimatized to the scanner environment in a “mock” scanner. This practice session consisted of 12 trials that presented equal payoffs across all decks. Participants were informed of this difference between the practice and experimental sessions. After completing the practice session, the researcher ensured that all participants understood the task through discussion with the participant and accompanying parent.

### Analysis of Performance Data

IGT net score was calculated for all 80 trials and separately for each of four blocks of 20 trials. Analysis of performance data was conducted using Bayesian analysis in JASP (v0*.*7.5.6; https://jasp-stats.org/)*.* Models were favored if Bayesian factor (BF)_10_ > 10 (35). Three-way analysis of variance (ANOVA) was used to compare groups on net score. To examine differences in learning over the course of the task, a 3 (group) × 4 (block) within-between repeated-measures ANOVA was performed on net scores separated into four blocks of 20 trials. Three separate 3 (group) × 2 (advantageous/disadvantageous, post-wins/post-loss, or stay/switch choices) within-between repeated-measures ANOVAs were used to examine potential group differences in reaction times.

### Computational Modeling

A hierarchical Bayesian analysis was implemented within hBayesDM (50). We first compared three established models using the Watanabe-Akaike Information Criterion (51). Details of the models and the model comparison are given in the Supplement.

The winning value-plus-perseverance model is a hybrid reinforcement learning and perseverance model. In this eight-parameter model, α represents feedback/magnitude sensitivity; λ represents loss-aversion; *c* represents choice consistency; *A* represents learning rate; *k* determines perseverance strength; εp and εn indicate loss/gain impact, respectively, on choice behavior (i.e., stay/switch tendency); and ω is the reinforcement learning weight (52). Group differences in mean parameter estimates were assessed by each parameter’s highest density interval (HDI), i.e., the range of parameter values that spans 95% of the distribution in a pairwise comparison 22, 50. Parameter estimates were considered to differ between groups if the HDI did not overlap zero 22, 50.

### MRI Image Acquisition

The fMRI images were acquired at King’s College London on a 3T General Electric Signa Horizon HDx MRI scanner (GE Healthcare, Milwaukee, WI) (see Supplement).

### fMRI Data Analysis

Data were analyzed using the nonparametric XBAM (v4.1) software (53), which overcomes many issues associated with parametric software packages (e.g., poor control of familywise error–corrected false positive clusterwise inference rates) 54, 55. Modeled events of interest included advantageous choices, disadvantageous choices, the anticipation period, win outcomes, and loss outcomes. fMRI analysis examined the decision phase (advantageous vs. disadvantageous choices), defined as the moment that the four decks are presented until choice execution (maximum: 6 seconds) and the outcome phase (wins vs. losses), during which the outcome appears on screen for 3 seconds (see Supplement for details).

For the group-level comparisons, analysis of covariance analyses with group as factor and head displacement in Euclidian 3D space and age as covariates were performed to compare groups. An examination of the effects of head displacement and age on brain activation is provided in the Supplement. The voxel-level threshold was set to *p <* .05; so as to maximize detection power, we used the highest threshold that we have shown empirically to give good type I error control at the cluster level under the null hypothesis using our permutation-based method 53, 54, 55, 56. A cluster-level *p* value threshold was computed from the data using our permutation-based method such that the final expected number of type I error clusters was < 1 (see Supplement).

Primary analyses were performed using regions of interest (ROIs) based on regions shown to play a role in IGT performance and/or to differ between ADHD and OCD groups or between patient groups and control subjects 7, 21, 24, 30, 40, 57, 58, 59, 60. A single ROI search space included the bilateral OFC, medial frontal gyrus, inferior frontal gyrus, insula, putamen, caudate, and nucleus accumbens. Regions were extracted from the Harvard-Oxford Atlas using FSL 61, 62. Within this search space, <1 false activated cluster was expected at *p <* .05 for voxel comparisons and *p <* .02 for cluster comparisons during decision and outcome phases.

Follow-up whole-brain comparisons of between-group differences were performed. For the between-group comparisons, <1 false activated cluster was expected at a cluster threshold of *p <* .004 for the choice phase and *p <* .0045 for the outcome phase.

To interpret the group differences in brain activation from the between-group analysis of covariance, statistical measures of blood oxygen level–dependent response for each participant were extracted from significant clusters, plotted, and subjected to pairwise (ADHD vs. OCD, ADHD vs. control subjects, OCD vs. control subjects) post hoc *t* tests (corrected for multiple comparisons for three groups using the least significance difference method). Within-group findings are presented in Supplemental Figures S5 and S6. Correlational analyses were performed between blood oxygen level–dependent response and performance and symptom measures (ADHD: Conners T; OCD: CY-BOCS) (see Supplement).

## Results

### Participant Characteristics

There were no group differences in age (Table 1). Groups differed on IQ (BF_10_ = 3.29, *F*_2,53_ = 4.48, *p =* .02), which was lower in patients with ADHD relative to control subjects (*p =* .007) and patients with OCD (*p =* .02), although all groups scored in the normal range for IQ, and no participant had IQ <85. Eight ADHD boys were medication naïve, and 8 were receiving stimulant medication. Sixteen boys with OCD were medication naïve, while 4 were being treated with selective serotonin reuptake inhibitor medication, and 1 patient was receiving risperidone augmentation treatment.

**Table 1:** Participant Characteristics and Behavioral Performance

	Control Subjects	ADHD	OCD	Statistics	Direction
*n*	20	16	20	—	
Age, Years	15.15 (1.99)	14.61 (1.87)	15.76 (1.43)	BF_10_ = 0.55, F_2,53_ = 1.88, *p* = .16	
IQ	119.7 (11.9)	107.6 (12.89)	117.7 (13.36)	BF_10_ = 3.29, *F*_2,53_ = 4.48, *p =* .02	C, OCD > ADHD
SDQ Hyperactivity/Inattention	2 (1.67)	8.5 (1.21)	4.4 (3.03)	Log(BF_10_) = 18.9, *F*_2,52_ = 39.1, *p <* .001	ADHD > OCD > C
CY-BOCS	—	—	22.32 (5.97)		
Conners T	—	80.94 (7.65)	—		
Net Score	10.45 (24.45)	−2.69 (18.7)	4.75 (17.4)	BF_10_ = 0.52, *F*_2,53_ = 1.18, *p =* .17	
Omissions %	0.75 (1.37)	2.56 (4.72)	0.75 (1.16)	BF_10_ = 0.84, *F*_2,53_ = 2.52, *p =* .09	
RT Advantageous, ms	1063.3 (443.2)	1133.0 (409.4)	1029.3 (220.2)	BF_10_ = 0.18, *F*_2,53_ = 0.36, *p =* .7	
RT Disadvantageous, ms	935.4 (323.2)	999.1 (285.8)	1041.8 (258.7)	BF_10_ = 0.23, *F*_2,53_ = 0.68, *p =* .51	
RT After Win, ms	935.2 (355.3)	1023.0 (319.3)	957.4 (238.9)	BF_10_ = 0.19, *F*_2,53_ = 0.38, *p =* .69	
RT After Loss, ms	1046.7 (351.6)	1119.7 (375.3)	1088.1 (246.2)	BF_10_ = 0.17, *F*_2,53_ = 0.23, *p =* .8	
RT Stay, ms	841.0 (396.9)	1161.1(655.6)	1040.4 (380.0)	BF_10_ = 0.63, *F*_2,53_ = 2.07, *p =* .14	
RT Shift, ms	1025.1 (355.2)	1057.3 (277.1)	1043.2 (255.1)	BF_10_ = 0.15, *F*_2,53_ = 0.05, *p =* .95	

Values are mean (SD) unless otherwise indicated.

ADHD, attention-deficit/hyperactivity disorder; BF, Bayesian factor; C, control subjects; CY-BOCS, Children's Yale-Brown Obsessive Compulsive Scale; OCD, obsessive-compulsive disorder; RT, reaction time; SDQ, Strengths and Difficulties Questionnaire.

### Performance Data

A 3 (group) × 4 (block) within-between repeated measures ANOVA showed no credible main effect of group in overall net score (BF_10_ = 0.56_,_ *F*_2,53_ = 1.18, *p =* .17), no main effect of block (BF_10_ = 0.35, *F*_3,159_ = 2.02, *p =* .11), and no group by block interaction effect (BF_10_ = 0.2, *F*_6,159_ = 1.59, *p =* .15). There were no group differences in reaction time or group by choice type interactions on reaction time (Table 1). Findings were unchanged after controlling for IQ, and there were no credible or significant correlations between symptoms (ADHD: Conners T; OCD: CY-BOCS) and net score or reaction time (all BF_10_ < 10, *p* > .05).

### Between-Groups Comparison of Value-Plus-Perseverance Model Parameters

Control subjects showed greater choice consistency (*c*) compared with patients with ADHD (95% HDI from 1.4 to 4.3, mean of HDI = 2.85; *t*_34_ = 28.27, *p < .*001) and patients with OCD (95% HDI from 1.7 to 4.5, mean of HDI = 3.1; *t*_38_ = 35.33, *p < .*001), as well as higher reinforcement learning weights (ω) than patients with ADHD (95% HDI from 0.02 to 0.57, mean of HDI = 0.3; *t*_34_ = 25.53, *p < .*001) and patients with OCD (95% HDI from 0.15 to 0.88, mean of HDI = 0.52, *t*_38_ = 33.56, *p < .*001). Patients with ADHD showed increased feedback sensitivity relative to control subjects (95% HDI from −1.99 to –0.02, mean of HDI = −1.01; *t*_34_ = 8.37, *p < .*001). There were no credible or significant correlations between symptoms (ADHD: Conners T; OCD: CY-BOCS) and model parameters (all BF_10_ < 10, *p* > .05). Complete tables of differential distributions and mean parameter estimates from the value-plus-perseverance model are presented in Supplemental Tables S1 and S2.

### Movement

The ANOVA analysis showed no group differences in mean Euclidean displacement (BF_10_ = 0.34, *F*_2,53_ = 1.2, *p =* .31).

### Between-Group Differences

For the decision phase, within the ROI, left VS underactivation during advantageous choices was shared in patient groups relative to control subjects, while vmOFC underactivation was disorder specific to patients with OCD. In the whole brain, ADHD and OCD patients shared abnormal activation in posterior cingulate cortex/precuneus/supplementary motor area relative to control subjects. In control subjects, this cluster was more active to disadvantageous choices, while in patients it was more active during advantageous choices.

**Figure 1:**
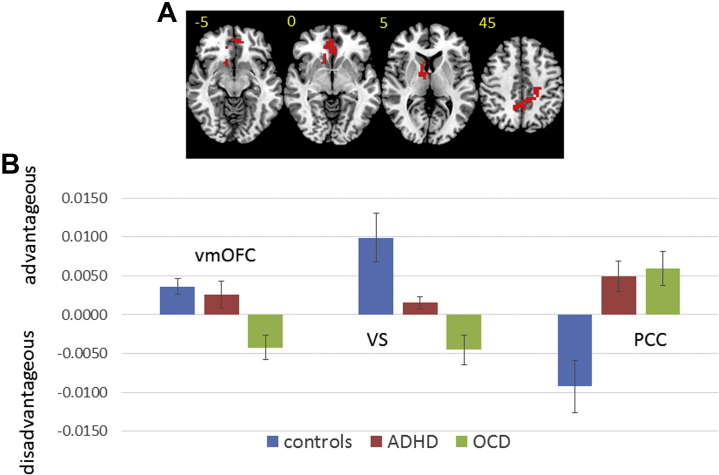
Analysis of covariance results for the between-group differences in brain activation for the contrast comparing advantageous and disadvantageous choices. **(A)** Axial slices for the group activation maps for the three groups. Red indicates regions showing significant between-group differences. Differences in the ventromedial orbitofrontal cortex (vmOFC) and ventral striatum (VS) were significant only within the region-of-interest search space. The difference in the posterior cingulate cortex (PCC) was significant in the whole brain. Talairach z coordinates are indicated for slice distance (in mm) from the intercommissural line. The right side of the brain corresponds to the right side of the image. **(B)** Bar chart showing mean blood oxygen level–dependent response for each group in each cluster. Control subjects = blue, attention-deficit/hyperactivity disorder (ADHD) = red, obsessive-compulsive disorder (OCD) = green.

**Figure 2:**
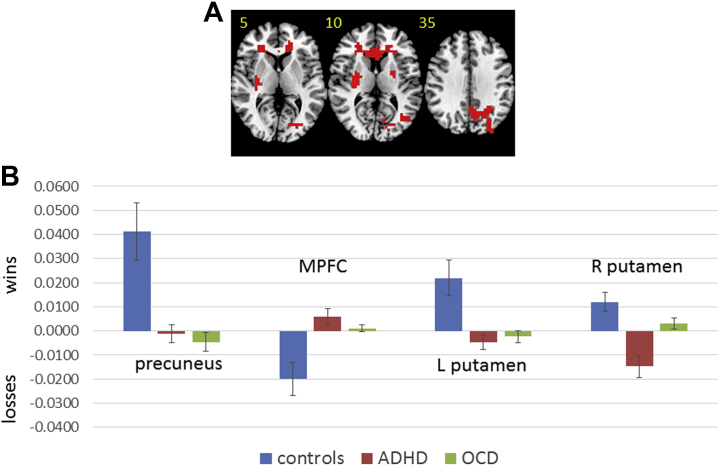
Analysis of covariance results for the between-group differences in brain activation for the contrast comparing win and loss outcomes. **(A)** Axial slices for the group activation maps for the three groups. Red indicates regions showing significant between-group differences. Differences in left and right putamen/caudate were significant only within the region-of-interest search space. Differences in the medial prefrontal cortex (MPFC) and precuneus were significant in the whole brain. Talairach z coordinates are indicated for slice distance (in mm) from the intercommissural line. The right side of the brain corresponds to the right side of the image. **(B)** Bar chart showing mean blood oxygen level–dependent response for each group in each cluster. Control subjects = blue, attention-deficit/hyperactivity disorder (ADHD) = red, obsessive-compulsive disorder (OCD) = green. L, left; R, right.

In the outcome phase, within the ROI, left putamen/caudate underactivation to wins was found in ADHD and OCD patients relative to control subjects. In right putamen/caudate, ADHD patients showed disorder-specific dysfunction relative to control subjects and patients with OCD. Patients with ADHD showed greater activation to losses, while control subjects showed greater activation to wins, and OCD patients showed little difference between conditions. In the whole brain, ADHD and OCD patients shared precuneus underactivation during wins relative to control subjects, as well as underactivation during losses in medial PFC (MPFC) (Table 2, Figures 1 and 2). After controlling for IQ, findings in the vmOFC, VS, and left putamen remained significant at the standard threshold (<1 error cluster). Findings in the posterior cingulate cortex/precuneus/supplementary motor area (*p =* .009), right putamen (*p <* .05), precuneus (*p =* .02), and MPFC (*p =* .03) remained significant only at relaxed cluster thresholds. An exploratory analysis using a whole-brain cluster threshold of *p <* .05 is included in the Supplement.

**Table 2:** ANCOVA Differences in Brain Activation Between Adolescents With ADHD and OCD and Healthy Comparison Adolescents

Brain Regions of Activation	BA	Tal Coord	Voxels	Cluster *p* Value	Pairwise *p* Values
Advantageous Choices > Disadvantageous Choices					

Control Subjects > ADHD and OCD Subjects					
L VS^a^		−11, 4, 4	35	.014	C vs. ADHD (*p =* .02) C vs. OCD (*p <* .001) ADHD vs. OCD (*p =* .08)
Control and ADHD Subjects > OCD Subjects					
vmOFC^a^	11	4, 41, −13	28	.011	C vs. ADHD (*p =* .61) C vs. OCD (*p <* .001) ADHD vs. OCD (*p =* .02)

Disadvantageous Choices > Advantageous Choices					

Control Subjects > ADHD and OCD Subjects					
SMA/PCC/precuneus	4/23/5	29, −26, 48	138	.003	C vs. ADHD (*p =* .001) C vs. OCD (*p <* .001) ADHD vs. OCD (*p =* .8)

Wins > Losses					

Control Subjects > ADHD and OCD Subjects					
L/R precuneus	19/7	36, −74, 37	185	.002	C vs. ADHD (*p =* .001) C vs. OCD (*p <* .001) ADHD vs. OCD (*p* = .77)
L putamen/caudate^a^		−22, 0, 9	44	.009	C vs. ADHD (*p =* .001) C vs. OCD (*p =* .001) ADHD vs. OCD (*p =* .4)
Control and OCD Subjects > ADHD Subjects					
R putamen/caudate^a^		22, −4, 9	48	.012	C vs. ADHD (*p <* .001) C vs. OCD (*p =* .08) ADHD vs. OCD (*p =* .001)

Losses > Wins					

Control Subjects > ADHD and OCD Subjects					
MPFC	32	−4, 48, 9	121	.004	C vs. ADHD (*p <* .001) C vs. OCD *(p =* .002) ADHD vs. OCD (*p =* .49)

ADHD, attention-deficit/hyperactivity disorder; ANCOVA, analysis of covariance; BA, Brodmann area; C, control subjects; L, left; MPFC, medial prefrontal cortex; OCD, obsessive-compulsive disorder; PCC, posterior cingulate cortex; R, right; SMA, supplementary motor area; Tal Coord, Talairach coordinates; vmOFC, ventromedial orbitofrontal cortex; VS, ventral striatum.

a Significant in region-of-interest search space.

## Discussion

The study investigated shared and disorder-specific neural and computational abnormalities during the IGT in adolescent ADHD and OCD. During decision making, both patient groups shared VS underactivation during advantageous choices, but patients with OCD showed disorder-specific underactivation in vmOFC relative to both control subjects and patients with ADHD. During the outcome phase, shared underactivation in patients to wins was found in the left putamen, and shared underactivation to losses was seen in the MPFC. Disorder-specific dysfunction was found in the right putamen/caudate, which was activated more to wins in control subjects but more to losses in patients with ADHD.

There were no significant group differences in net scores, unlike in previous work in both disorders in adolescent samples 63, 64. This may be due to a highly medicated sample 65, 66, 67, due to the relatively high IQ across all groups that may have compensated for suboptimal performance in patient groups 68, 69, or due to our use of a shortened version of the IGT. Indeed, the previous report of performance differences on the IGT in adolescents with OCD found significant differences only on the final 20 trials (63). Computational modeling and neuroimaging may be more sensitive measures of abnormalities in reward and decision-making brain networks than net score, as normal overall performance may be maintained despite underlying neural dysfunction and more subtle cognitive abnormalities 35, 60.

The computational modeling showed that patient groups made more exploratory choices from decks with lower expected rewards and were more likely to make decisions based on feedback from the most recent trial rather than based on reward expectancies formed over successive trials relative to control subjects (although all groups tended to make decisions based on reinforcement contingencies).

Previous work has reported reduced choice consistency in ADHD (13). ADHD symptoms and neuropsychological performance patterns, including impaired choice consistency, may be explained in terms of low neural gain, that is, an altered balance between the neural signals supporting the chosen or optimal goals, behaviors, and attentional targets and competing signals that support alternative actions and cognitions, in which the goal-directed signals are insufficiently strong and the competing signals are poorly suppressed, thus resulting in behavioral and attentional instability (70).

In OCD, increased exploration of nonoptimal decks may be related to greater intolerance of uncertainty, reduced confidence in memories and decisions, and increased need for information sampling in the disorder 71, 72, 73, 74. In other words, OCD patients may have decreased confidence in their assessment of deck expectancies and their memories of previous outcomes, with this underlying an increased tendency to recheck the alternative decks (75).

A disorder-specific finding was that ADHD patients alone showed heightened feedback sensitivity. Although sensitivity to the magnitude of deck outcomes is important for IGT performance, increased sensitivity may lead to a tendency to chase large wins on the disadvantageous decks. Heightened feedback sensitivity on the IGT has been associated with impulsivity related behaviors and disorders 22, 76, 77, which are more prevalent in ADHD 78, 79, 80, and the current findings provide further support for a relationship between feedback sensitivity and impulsivity related disorders.

In the brain, the VS was underactive during advantageous choices in patient groups relative to control subjects. The VS responds to reinforcers including monetary reward 25, 26 and contributes information about the motivational properties and magnitudes of available rewards, initially biasing decision making toward impulsive, immediate, or larger but riskier rewarding actions 81, 82. During learning, dopamine cell responses within the VS shift from primary reinforcers to cues or behaviors that predict rewarding outcomes 83, 84, 85. VS responses during advantageous choices in control subjects may represent the positive expected values for the advantageous decks. In other words, in control subjects robust mesolimbic signaling during decision making may have guided choices toward the optimally rewarding decks.

In ADHD, the dopamine response in the VS to previously neutral cues or behaviors that are now associated with reward is hypothesized to be disrupted, meaning that the motivational features and underlying VS activation that these cues take on in control subjects may be decreased in ADHD 12, 15. In the IGT, weaker representations of deck reinforcement history within the VS may underlie impairments in patients in selecting decks associated with the highest expected values.

In OCD, findings of VS underactivation are consistent with previous reports of reduced VS response to cues that predict reward 10, 11, 42. Patients with OCD show increased VS and dorsal striatal responses during symptom provocation and habitual responding 86, 87, 88, enlarged basal ganglia structure 57, 89, and altered activity/connectivity at rest 90, 91. Alterations in VS-mediated salience, habit, and motivation functions may underlie an imbalance between competing unrewarded OCD behaviors and goal-related behaviors in the disorder, with VS hypoactivation during decision making underlying deficits in representing outcome contingencies and an impaired selection of goal-related choices and behaviors.

As hypothesized, adolescents with OCD showed disorder-specific underactivation in vmOFC during advantageous choices. The vmOFC is closely interconnected with VS and is a key structure for flexible emotional learning and decision making 21, 24, 28. The vmOFC is highly implicated in OCD 9, 39, 57, 86, 91. The current results extend previous findings by suggesting a role for vmOFC dysfunction in decision making under ambiguity in the disorder. The findings also extend our comparative meta-analysis of voxel-based morphometry studies and fMRI studies of inhibitory control tasks that showed disorder-specific underactivation and reduced structure in vmOFC in OCD relative to ADHD (40), as well as our recent finding of disorder-specific vmOFC underactivation during temporal discounting in adolescents with OCD relative to adolescents with ADHD and control subjects (7).

During outcome processing, an unexpected finding was that unlike in some 44, 92, but not all 93, 94, previous studies using the monetary incentive delay task, patients with ADHD did not exhibit increased activation to wins in vmOFC or VS, and instead showed disorder-specific increased activation to losses in the right putamen/caudate and shared underactivation in the left putamen/caudate to wins. A lack of increased reactivity to wins may reflect differences between the monetary incentive delay task and IGT. For instance, in the monetary incentive delay task contingencies between cues and reward outcomes do not need to be learned, whereas outcome evaluation in the IGT is important for learning the outcomes associated with each deck, and qualitatively different orbito-fronto-striatal signaling may be involved in passive reward receipt and active outcome evaluation (29). The disorder specificity of the right putamen underactivation is interesting in view of previous meta-analytic findings of disorder-specific reduced right putamen gray matter volume and activation in ADHD relative to OCD in voxel-based morphometry studies and fMRI studies of inhibitory control (40). The findings extend evidence for disorder-specific right striatal underactivation in ADHD relative to OCD during inhibitory control to the domain of reward-based decision making.

The whole-brain analysis revealed that patient groups shared reduced activation to losses in MPFC. Underactivation to losses in the MPFC is in line with previous findings of reduced MPFC localized feedback–related negativity to monetary loss in ADHD patients (95). Reduced MPFC activation to losses is in line with fMRI studies of reversal learning in OCD, which report decreased activation in MPFC and adjacent OFC when participants learn to shift responses based on negative feedback 27, 96, 97, 98, as well as with findings of reduced MPFC gray matter volume and reduced MPFC activation during inhibitory control 40, 57. Findings support a shared blunting of neural responses during outcome processing in adolescent ADHD and OCD.

Limitations include, first, the fact that 50% of patients with ADHD were receiving stimulant medication, while 20% of patients with OCD were receiving antidepressant medication and one patient with OCD was receiving risperidone. There were too few unmedicated patients to conduct a subgroup analysis. These medication treatments may alter functioning in the dopaminergic mesolimbic pathways responsible for decision making and outcome processing, with stimulant medications increasing striatal dopamine in ADHD, but selective serotonin reuptake inhibitors and risperidone reducing dopaminergic functioning in OCD 99, 100. Although patients with ADHD underwent a 48-hour washout period, there is meta-analytic evidence for a normalization of frontostriatal activation and alterations in dopaminergic functioning with chronic stimulant treatment in patients with ADHD 38, 101. Second, groups differed on IQ, which was lower in patients with ADHD relative to the other groups. However, lower IQ is typical for this population and all groups scored in the normal range for IQ (102). Third, structured interviews to assess common comorbidities including anxiety, mood, and autism spectrum disorders in patients and undiagnosed conditions in control subjects were not performed, and owing to their common co-occurrence, subclinical comorbid ADHD and OCD symptoms might have been present in the patient groups. However, participants were considered by a consultant psychiatrist to be free of comorbidities after clinical assessment. Fourth, brain activation during the outcome phase may have been contaminated by brain activation from the anticipation phase owing to hemodynamic delay and a lack of jitter between the two phases in the task design. Fifth, findings are not generalizable to girls with ADHD or OCD. Last, although the sample size is typical for the adolescent ADHD and OCD fMRI literature, future work should aim to confirm these findings in larger samples.

In summary, this is the first study to compare decision making under ambiguity in adolescent ADHD and OCD using fMRI and computational modeling. Findings of shared choice consistency impairments and smaller reinforcement learning weights, as well as findings of shared VS underactivation during advantageous choices, suggest impairment in both disorders in representing and utilizing learned reward expectancies during decision making. Findings of reduced sensitivity to outcomes in MPFC and left putamen suggest shared alterations in outcome processing when outcomes must be used to guide future behavior. Disorder-specific dysfunction in the vmOFC in OCD and in the right putamen in ADHD parallel previous, similar multimodal meta-analytic findings in voxel-based morphometry studies and fMRI studies of inhibitory control, indicating a possible preservation of disorder-specific markers across tasks and modalities.
