# Supplementary material for: Frontostriatal Dysfunction During Decision Making in Attention-Deficit/Hyperactivity Disorder and Obsessive-Compulsive Disorder
Source: Biol Psychiatry Cogn Neurosci Neuroimaging. 2018 Aug;3(8):694–703. doi: 10.1016/j.bpsc.2018.03.009 (PMC6278892; doi:10.1016/j.bpsc.2018.03.009)
Supplement: Supplemental Material [file mmc1.pdf]

Fronto-striatal Dysfunction During Decision-making in Attention-Deficit/Hyperactivity Disorder and Obsessive-Compulsive Disorder

Supplemental Information

Supplementary Methods

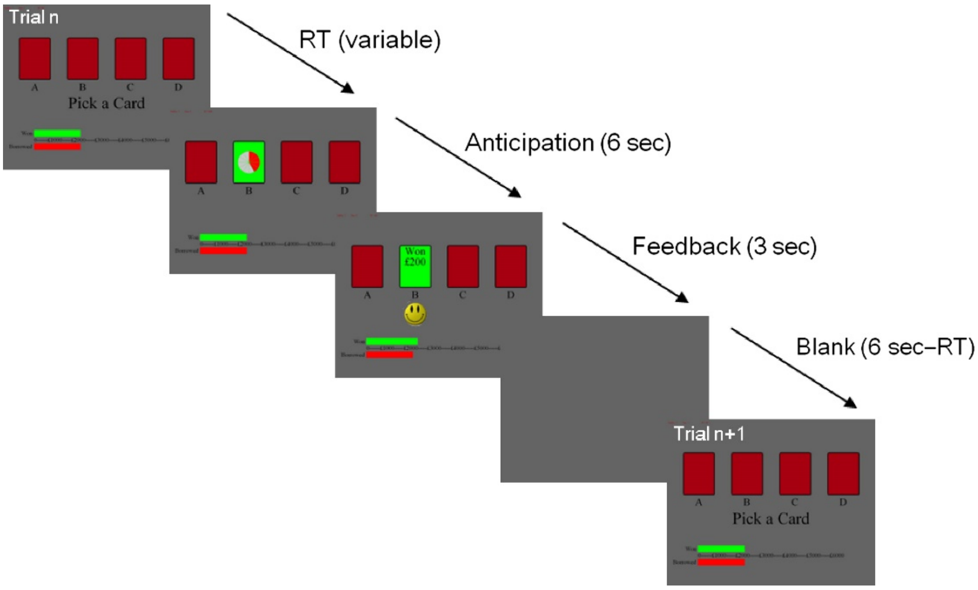

Supplementary Figure S1. Schematic representation of the Iowa gambling task (IGT). On each trial of the IGT, participants chose from one of four decks by pressing the spatially corresponding button on an MR-compatible button box. The decision phase was followed by an anticipation phase (6 s) before the outcome evaluation phase (3 s) displayed the outcome of the decision (win/loss and magnitude). Each trial culminates with a blank screen that took the total trial duration to 15 s. The loan (red bar) and the current running total (green bar) were presented at the bottom of the task display (1-3).

Computational Modelling

Prospect Valence Learning Models

For all three models, outcome evaluation is assessed according to the non-linear prospect utility function which takes into account a diminishing sensitivity to increasing levels of magnitude, as well as a different sensitivity to loss and gain outcomes (4, 5). The utility u(t) on trial *t* of each outcome *x(t)* is expressed as:

$$u(t) = \begin{matrix} x(t)^{a} if x(t) \geq 0 \\ -\lambda |x(t)|^{a} if x(t) < 0 \end{matrix} \qquad (1)$$

The α (0<α<2) parameter determines the shape of the utility function and represents feedback sensitivity. A higher value represents higher sensitivity to the relative magnitudes of outcomes. The λ (0<λ<10) parameter represents loss-aversion, which determines the participant's sensitivity to losses compared to gains. As λ approaches 0, losses are experienced as neutral events. For λ=1, losses and gains have the same impact. When λ>1, losses have greater impact than gains on the subjective utility of an outcome, i.e. the participant prefers to select the alternative with a decreased probability of losses despite lower expected gains.

The parameter *A* (0<*A*<1) represents the learning rate and determines how much past expectancy is discounted. A high learning rate indicates that the recent outcome has a large influence on the expectancy of the chosen deck, while a low learning rate indicates the opposite. The prospect valence models are identical except that they use different learning rules (4). In the delta model, expectancies are recency-weighted averages of the rewards received thus far for each deck. A Rescorla-Wagner (6) updating equation updates the

expected value of the chosen deck on each trial. Expectancies of the other decks are unchanged until chosen on a different trial.

$$E_j(t+1) = E_j(t) + A \cdot \delta_j(t) \cdot \left(u(t) - E_j(t)\right) \qquad (2)$$

Ej(t) refers to the expectancy for deck *j* on trial *t*. In this model, *A* is a recency parameter that indicates the weight given to recent outcomes when updating expectancies for the chosen deck. The variable *δj(t)* is a dummy variable, which is 1 if deck *j* is chosen or else is equal to 0.

In the decay model, a decay learning rule (7) is used in which expectancies of all unchosen decks are discounted on each trial, and the expectancy of the chosen deck is updated by the current outcome utility. In this model, *A* is a decay parameter, which indicates the degree to which expectancy is discounted:

$$E_j(t+1) = A \cdot E_j(t) + \delta_j(t) \cdot u(t) \qquad (3)$$

Next, the PVL models assume that the participants' choices on the trial t+1 are guided by the expectancies of each deck. This is formalized using a softmax function with an inverse-temperature parameter:

$$Pr[D(t+1) = j] = \frac{e^{\theta \cdot E_j(t+1)}}{\sum_{k=1}^{4} e^{\theta \cdot E_k(t+1)}} \qquad (4)$$

This function contains a trial-independent sensitivity parameter θ that indexes the extent to which trial-by-trial choices match the expected deck utilities, and which is set by the fourth model parameter, which is *c* ($0 \leq c \leq 5$) or response consistency. Large values of *c* indicate a tendency to select decks with the highest learned expectancies (exploitation), whereas small values indicate a tendency explore decks with lower expected utilities (exploration).

Value Plus Perseverance Model

Evidence suggests that participants often use a WSLS strategy during IGT (8). In other words, participants sometimes adopt a perseverative strategy that uses information only about the most recent outcome for the most recently chosen deck. A hybrid Value Plus Perseverance (VPP) model was developed combining the PVL-Delta learning rule and WSLS heuristic (8). This model assumes that individuals track both expectancies *(Ej(t))* and perseverance strengths *(Pj(t))*; expectancies are computed using the learning rule of the PVL-Delta model, and three additional perseverance parameters are included:

$$P_j(t+1) = \begin{matrix} k \cdot P_j(t) + \varepsilon_p if x(t) \geq 0 \\ k \cdot P_j(t) + \varepsilon_n if x(t) < 0 \end{matrix} \qquad (5)$$

k (0<*k*<1) determines how much perseverance strengths of all (including unselected) decks decay on each trial, and εp and εn indicate loss/gain impact, respectively, on choice behaviour. Positive values reflect a tendency to persevere on the same deck, while negative values indicate a tendency to switch decks on the next trial. Overall value, *Vj(t+1)* is the weighted sum of *Ej(t+1)* and *Pj(t+1)*:

$$V_j(t+1) = \omega \cdot E_j(t+1) + (1-\omega) \cdot P_j(t+1) \qquad (6)$$

ω is the reinforcement learning (RL) weight (0<ω<1); a low ω indicates the subject relies less on RL/more on perseverance. Choice probability was again computed using the softmax function, but with *Vj(t+1)*:

$$Pr[D(t+1) = j] = \frac{e^{\theta \cdot V_j(t+1)}}{\sum_{k=1}^{4} e^{\theta \cdot V_k(t+1)}} \qquad (7)$$

Model Comparison

Posterior inference for all models was performed via Markov Chain Monte Carlo (MCMC) sampling implemented in RStan (v2.1.063) (9) (http://mc-stan.org/interfaces/rstan). RStan uses a specific probabilistic sampler called Hamiltonian Monte Carlo (HMC) to sample from the posterior distribution. A total of 3000 samples per chain were drawn after 1000 burn-in samples with 4 chains (10, 11). All model parameters of all models had R^ values around 1.00, indicating that chains converged to the target distributions (10). Post-hoc model comparison was conducted using Wanatabe-Akaike Information Criterion (WAIC) (12). This index is obtained by computing the summed point-wise log-likelihood per participant, accounting for the fact that in the IGT, choices on a given trial are dependent on previous choices (13). Smaller WAIC scores denote better model-fit, and overall fit was assessed by adding WAIC scores from each group for each model (12).

In line with previous work, across all participants the VPP model (WAICtotal=11337.88) provided the best model-fit relative to the other two models (PVL-Decay WAICtotal=11589.93; PVL-Delta WAICtotal=11925.39). This model was also the best fit within control (VPP WAIC=3670.54; PVL-Decay WAIC=4076.48; PVL-Delta WAIC=4135.61), ADHD (VPP WAIC=3107.35; PVL-Decay WAIC=3355.58; PVL-Delta WAIC=3392.5) and OCD (VPP WAIC=3851.94; PVL-Decay WAIC=4202.03; PVL-Delta WAIC=4270.9) groups separately. Consequently, the model parameters from the winning VPP model were used to compare groups.

fMRI Image Acquisition

The fMRI images were acquired at King's College London, Institute of Psychiatry's Centre for Neuroimaging Sciences on a 3T General Electric Signa Horizon HDx MRI scanner (GE

Healthcare, UK) using the body coil for radio frequency transmission and a quadrature birdcage headcoil for reception. In each of 22 non-contiguous planes parallel to the anterior–posterior commissure, 480 T2*-weighted MR images depicting BOLD contrast covering the whole brain were acquired with echo time TE=30 ms, TR=1.5 s, flip angle=60°, in-plane voxel size=3.75 mm, slice thickness=5.0 mm, slice skip=0.5 mm). A whole-brain high resolution structural scan (inversion recovery gradient echo planar image) used for standard space normalisation was also acquired in the inter-commissural plane with TE=40 ms, TR=3 s, flip angle=90°, number of slices: 43, slice thickness=3.0 mm, slice skip=0.3 mm, in-plane voxel size=1.875 mm, providing complete brain coverage. As collected data in patients with OCD included only adolescents aged 12-18 years old, data from control and ADHD groups for older (adults) and younger (pre-adolescents) age ranges were excluded so that groups could be matched on age. One patient with OCD was excluded due to poor quality fMRI data. Participants completed other tasks during the same session, which have been published elsewhere (1-3, 14-23).

XBAM Analysis

In contrast to theory-based inference, XBAM (24-26) minimizes assumptions and uses median based statistics which controls for outliers. The test statistic is computed by standardizing for individual differences in residual noise before performing second-level analysis using permutation tests. XBAM employs a mixed-effects method that has been recommended following analysis of the validity and impact of theory-based inference in fMRI (27).

Following motion correction, global detrending, spin-excitation history correction and smoothing (full-width at half-maximum (FWHM) 7.2 mm), the residual effects of motion were regressed out from the time series (using the estimated motion parameters) before a

standard general linear modelling approach was used to obtain estimates of the response size (beta) to each of the events of interest (advantageous choices, disadvantageous choices, anticipation period, win outcomes, loss outcomes). The anticipation and outcome phases are likely not entirely separable, due to the lack of jitter between the two conditions in the task design. Moreover, it is important to note that on all trials the probability of winning was 50/50, which means that there is no equivalent to a “wins” versus “loss” anticipation contrast in our version of the IGT, as has been examined in tasks such as the monetary incentive delay task (28). Therefore we did not examine the anticipation phase in the current study, and must note as a limitation that brain activation during the outcome phase may be contaminated by brain activation from the anticipation phase due to the haemodynamic delay.

Time series analysis for each subject was conducted using wavelet-based resampling (29). We convolved the main experimental conditions with 2 Poisson model functions (peaking at 4 and 8 s). We then calculated the weighted sum of these 2 convolutions that gave the best fit (least-squares) to the time series at each voxel. A goodness-of-fit statistic (SSQ ratio) was computed at each voxel consisting of the ratio of the sum of squares of deviations from the mean intensity value due to the model (fitted time series) divided by that of the squares due to the residuals (original time series minus model time series) (29).

The appropriate null distribution for assessing significance of any given SSQ ratio was established using a wavelet-based re-sampling method and applying the model-fitting process to the resampled data. The aim was to achieve a global (image-wide) permutation based threshold for forming clusters at $p<0.05$ at the first stage of cluster analysis. We did this by permuting 20 times per voxel and combining permutations over the whole brain, resulting in 20 null parametric maps of SSQ ratios for each subject, which were combined to give the

overall null distribution of SSQ ratio (26, 29). As there are approximately 50,000 intra-cerebral voxels in our analysis, this means that we used around 1,000,000 combined permutations to form a global threshold for the first stage of our cluster analysis (18, 29). This same permutation strategy was applied at each voxel to preserve spatial correlation structure in the data. Individual SSQ ratio maps were then affine transformed into standard space by normalising onto a Talairach template (30).

For the group-level comparisons, group activation maps were produced for each contrast by calculating the median observed SSQ-ratio over all subjects at each voxel in standard space and testing them against the null distribution of median SSQ-ratios computed from the identically transformed wavelet re-sampled data (24). ANCOVA analyses with group as factor and head displacement in Euclidian 3-D space and age as covariates were performed to compare groups. The necessary combination of voxel and cluster level thresholds was not assumed from theory but rather was determined by direct permutation for each dataset (26). The voxel-level threshold was first set to $p<0.05$ to give maximum sensitivity and to avoid Type II errors, as in order to maximize detection power we used the highest threshold that we have shown empirically to give good type I error control at cluster level under the null hypothesis using our permutation-based method (24, 26, 29). A cluster-level threshold was computed from the data for the resulting three-dimensional voxel clusters such that the final expected number of type I error clusters was <1. Cluster mass rather than a cluster extent threshold was used to minimize discrimination against possible small, strongly responding foci of activation (26).

Supplementary Results

Effects of Age and Motion on Brain Activation

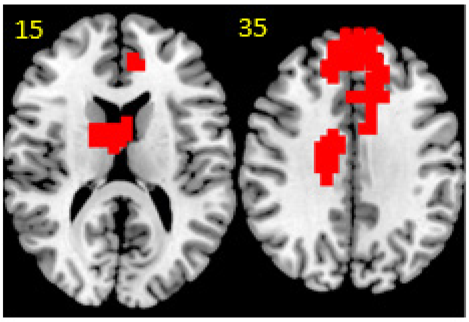

Supplementary Figure S2. Bilateral MPFC/dACC/SMA/caudate (Peak Talairach Coordinates: -4,52,31, voxels=233, *p*=0.01) regions showing a significant positive correlation between head displacement in Euclidian 3-D space and brain activation during outcome processing (wins>losses) across all participants. Thresholded at p<0.05 for voxel and expected number of type I error clusters was <1.

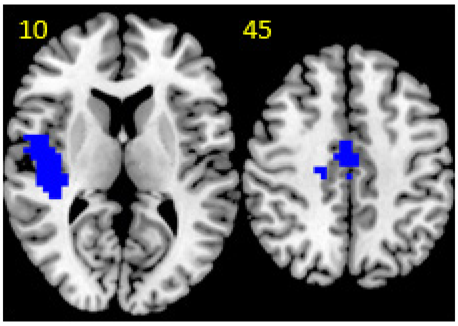

Supplementary Figure S3. Regions in left insula/STG/postcentral gyrus (Peak Talairach Coordinates: -40,-7,-2, voxels=175, p=0.005) and dACC/SMA/MPFC/PCC/SPL (Peak Talairach Coordinates: 4,-4,31, voxels=329, p=0.003) showing a significant negative correlation between age and brain activation during decision making (advantageous>disadvantageous choices) across all participants. Thresholded at p<0.05 for voxel and expected number of type I error clusters was <1.

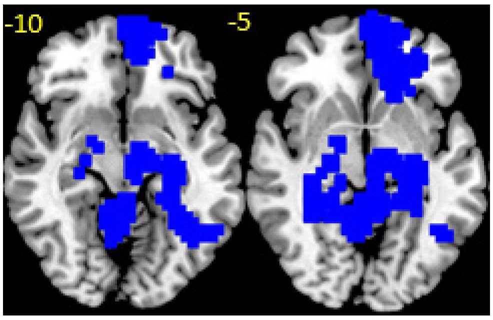

Supplementary Figure S4. Bilateral cerebellum/thalamus/right occipital lobe/ITL (Peak Talairach Coordinates: 11,-74,-29, voxels=495, *p*=0.003) and right vmOFC/MPFC/IFG (Peak Talairach Coordinates: 29,52,-2, voxels=256, p=0.004) regions showing a significant negative correlation between age and brain activation during outcome processing (wins>losses) across all participants. Thresholded at $p<0.05$ for voxel and expected number of type I error clusters was <1.

Exploratory Brain-Behaviour and Brain-Performance Correlations

Statistical BOLD response from regions that showed significant group differences were extracted, and correlated with task performance (net score, and VPP parameters shown to differ between groups: *c,* ω, α) and symptoms scores (ADHD: Conner's T; OCD: CY-BOCS).

Within controls, net score was correlated with activation in vmOFC (BF_{10}=11.7, $r(20)=0.61$, $p=0.005$) during the choice phase, such that greater activation during advantageous relative to disadvantageous choices was associated with a greater proportion of advantageous choices.

In addition, using NHST, in controls VS activation was correlated with net score ($r(20)=0.49$, $p=0.03$). Moreover, VS ($r(20)=0.45$, $p<0.05$) and vmOFC ($r(20)=0.53$, $p=0.02$) activation was correlated with *c*, such that greater activation was associated with greater choice consistency.

In ADHD and OCD patients, there were significant correlations between SMA/PCC/precuneus activation and net score. In patients with ADHD, greater activation to disadvantageous choices was associated with a higher net score ($r(16)=-0.57$, $p=0.02$), while in OCD patient greater activation to advantageous choices was associated with a higher net score ($r(20)=0.58$, $p=0.01$). However, crucially, for all these findings $BF_{10}<10$, suggesting weak evidence for the tested model over the null hypothesis. Therefore these results should be interpreted with caution.

There were no credible or significant correlations between symptoms and brain activation within either ADHD or OCD patient groups (all $BF_{10}<10$, $p>0.05$).

Supplementary Table S1. Parameter estimates from the VPP model

Parameter	HC (N=20) mean	ADHD (N=16) mean	OCD (N=20) mean
Learning rate (*A*)	0.01	0.05	0.14
Feedback sensitivity (α)	0.14	1.29	0.94
Choice sensitivity (*c*)	3.22	0.66	0.39
Loss aversion (λ)	0.18	2.34	4.79
Loss impact (ε_p)	-6.43	-7.04	-6.51
Gain impact (ε_n)	-3.87	-4.94	-3.3
Perseverance decay rate (*k*)	0.41	0.47	0.42
Reinforcement learning weight (ω)	0.99	0.75	0.51

Abbreviations: ADHD, Attention-Deficit/Hyperactivity Disorder; HC, healthy controls; OCD, Obsessive-Compulsive Disorder; VPP, value-plus-perseverance.

Supplementary Table S2. Highest density intervals for two-way comparisons

Parameter	95% HDI of MCMC	
	HC vs. ADHD	
A	-0.124329631	0.009118273
α	-1.98504670	-0.02234436
c	1.403504	4.304253
λ	-8.2034198	0.5380411
ε_p	-10.40527	11.64826
ε_n	-7.115025	10.562547
k	-0.22995921	0.08383159
ω	0.01793361	0.57137645
	HC vs. OCD	
A	-0.4283741	0.0170836
α	-1.96324296	0.09953476
c	1.702021	4.499517
λ	-9.39909698	0.02869173
ε_p	-11.18040	10.95115
ε_n	-9.012880	7.633389
k	-0.2080696	0.2080198
ω	0.1466798	0.8833930
	ADHD vs. OCD	
A	-0.4164726	0.1212036
α	-1.224132	1.719832
c	-0.1712017	0.7308317
λ	-9.786166	4.741136
ε_p	-11.02590	10.76896
ε_n	-10.580384	6.735364
k	-0.1216061	0.2647576
ω	-0.2311466	0.7312512

Abbreviations: α, outcome sensitivity; *A*, learning rate; ADHD, Attention-Deficit/Hyperactivity Disorder; c, consistency/choice sensitivity; ε_p /ε_n, impact of gain/loss, respectively, on perseverance behaviour; HC, Healthy Control; HDI, highest density interval; *k*, perseverance decay rate; λ, loss aversion; MCMC, Markov Chain Monte Carlo sampling; OCD, Obsessive-Compulsive Disorder; ω, reinforcement learning weight.

(A) Healthy control boys

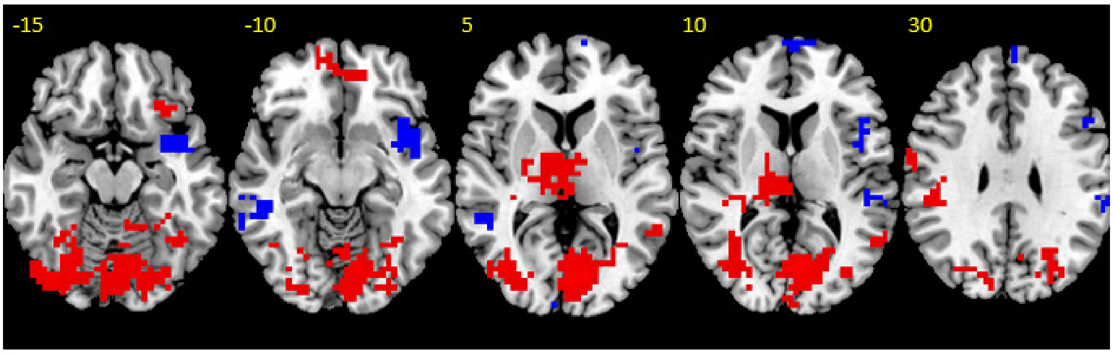

(B) ADHD boys

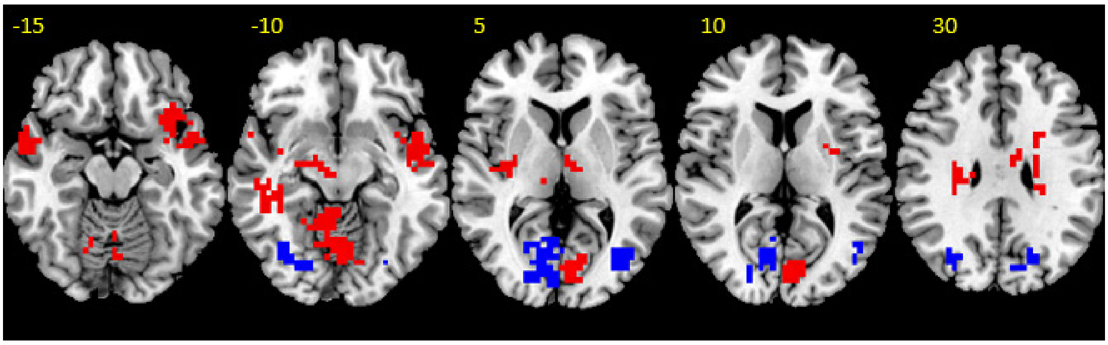

(C) OCD boys

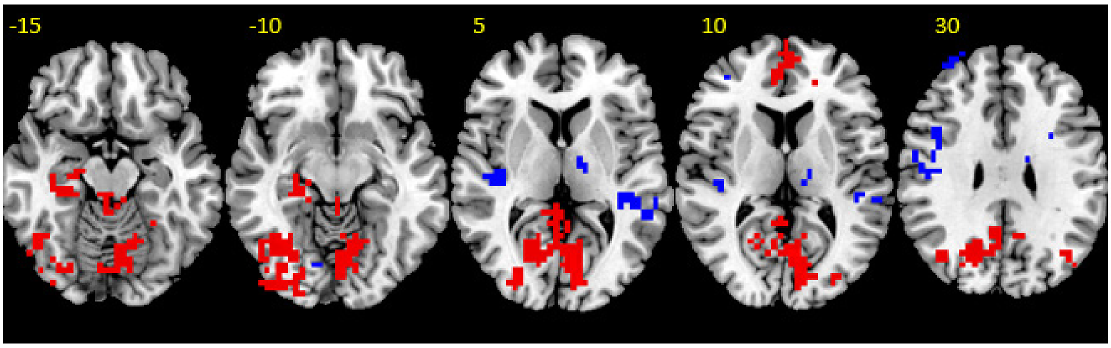

Supplementary Figure S5. Within-group maps for choice phase (disadvantageous versus advantageous). Group activation maps. Axial slices showing within-group brain activation for the contrasts of advantageous-disadvantageous choices (red) and disadvantageous-advantageous choices (blue). (A) healthy controls, (B) ADHD boys (C) OCD boys. Talairach z-coordinates are indicated for slice distance (in mm) from the intercommissural line. The right side of the brain corresponds to the right side of the image. Data presented at voxel threshold $p<.05$ and cluster threshold $p<0.05$.

(A) Healthy control boys

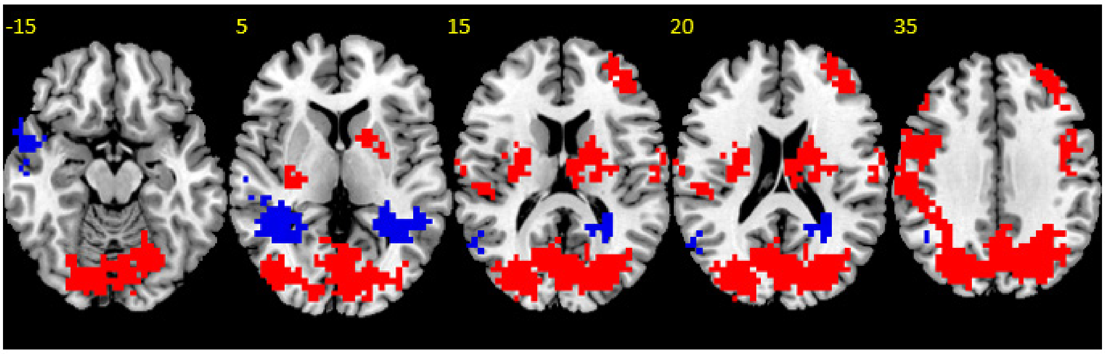

(B) ADHD boys

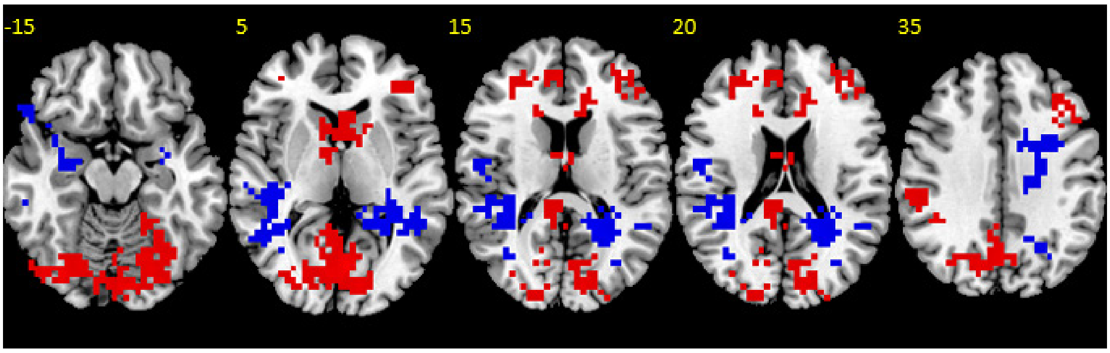

(C) OCD boys

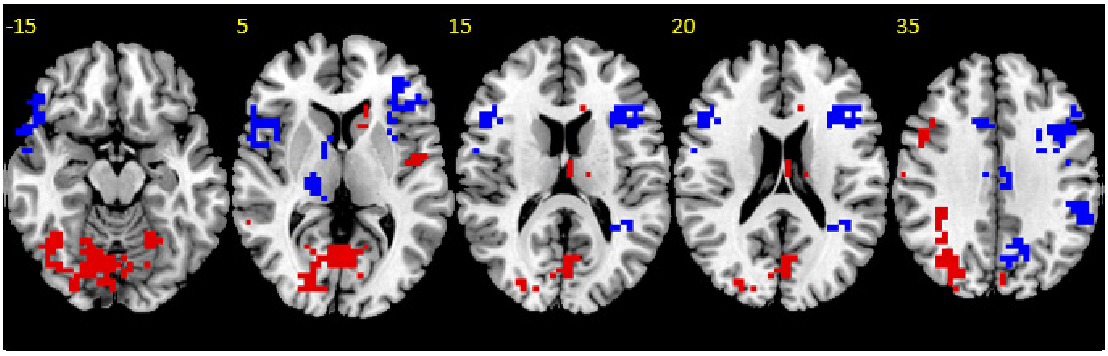

Supplementary Figure S6. Within-group maps for outcome phase (wins versus losses). Group activation maps. Axial slices showing within-group brain activation for the contrasts of win-loss outcomes (red) and losses-wins choices (blue). (A) healthy controls, (B) ADHD boys (C) OCD boys. Talairach z-coordinates are indicated for slice distance (in mm) from the intercommissural line. The right side of the brain corresponds to the right side of the image. Data presented at voxel threshold $p<.05$ and cluster threshold $p<.05$.

Supplementary Table S3. ANCOVA differences in brain activation between adolescents with ADHD and OCD and healthy comparison adolescents at a liberal whole-brain cluster threshold (p<.05).

Brain regions of activation	BA	Peak Talairach Coordinates	Voxels	Cluster p-value
**Advantageous>disadvantageous choices**				
**Controls > ADHD, OCD**				
L VS/thalamus		-11,4,4	43	.014
R occipital lobe	18	36,-81,-7	21	.045
L occipital lobe	19	-29,-70,4	25	.033
R occipital lobe	19	25,-59,4	27	.036
L cerebellum		-22,-78,-24	20	.029
L cerebellum		-51,-70,-29	30	.021
R cerebellum		25,-70,-24	16	.044
**Controls, ADHD > OCD**				
vmOFC	11	4,41,-13	28	.02
L DLPFC/IFG	10,46,45	-36,44,15	22	.022
**Disadvantageous > advantageous choices**				
**Controls > ADHD, OCD**				
aPFC	10	4,70,9	24	.03
R STG/insula	38,21	47,7,-13	22	.026
R pallidum/thalamus		18,-11,-2	33	.03
R STG/SMG	42,40	65,-30,20	18	.03
L & R precentral gyrus/postcentral gyrus/PCC/precuneus	4,5,6	2,-26,48	138	.029
**Wins>losses**				
**Controls >ADHD, OCD**				
R DLPFC/IFG	9,46,44,45	36, 52, 26	46	.022
L dACC/SMA/precentral gyrus/postcentral gyrus	6,4	-18-19,59	20	.03
L putamen		-22,0,9	44	.013
L precentral gyrus	6,4	-40,0,26	17	.043
L superior parietal/inferior parietal, SMG	7,40	-20, -61, 61	71	.009
R precuneus/superior parietal/occipital lobe	7,18,19	36, -74, 37	185	.002

Brain regions of activation	BA	Peak Talairach Coordinates	Voxels	Cluster p-value
L occipital lobe/angular gyrus	19,18,37,39	-37, -77, -3	45	.033
**Controls, OCD >ADHD**				
R putamen/caudate		22,-4,9	50	.016
**Losses>wins**				
**Controls > ADHD, OCD**				
L OFC	11	-25,33,-40	17	.032
MPFC	32,24,25,9	-4,48,9	121	.005
dACC	32,6,8	-14,19,42	9	.05
L STG/MTG	22,21	-47,-26,-7	19	.049
**Controls, ADHD > OCD**				
R caudate tail/hippocampus, parahippocampus/MTG/occipital lobe	37,41,19	33,-30,4	74	.02
**ADHD> Controls, OCD**				
R dACC/SMA/precentral gyrus/postcentral gyrus	6,4	11,-15,59	21	.032

Abbreviations: ADHD, Attention-Deficit/Hyperactivity Disorder; aPFC, anterior prefrontal cortex; BA, Brodmann area; dACC, dorsal anterior cingulate cortex; DLPFC, dorsolateral prefrontal cortex; IFG, inferior frontal gyrus; MTG, middle temporal gyrus; PCC, posterior cingulate cortex; OCD, Obsessive-Compulsive Disorder; OFC, orbitofrontal cortex; SMA, supplementary motor area; SMG, supramarginal gyrus; STG, superior temporal gyrus; vmOFC, ventromedial orbitofrontal cortex; VS, ventral striatum.

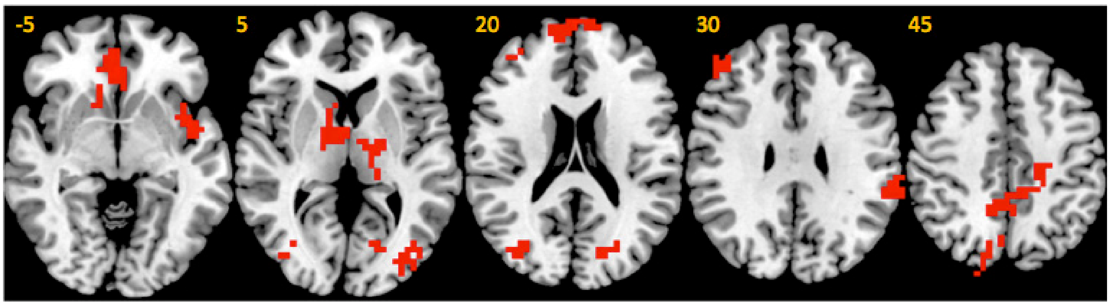

Supplementary Figure S4. Exploratory ANCOVA results for the between-group differences in brain activation for contrast comparing advantageous and disadvantageous choices at a liberal whole-brain cluster threshold (p<.05). Red indicates significant between-group differences in activation between adolescents with ADHD, adolescents with OCD, and healthy comparison adolescents. Talairach z-coordinates are indicated for slice distance (in mm) from the intercommissural line. The right side of the brain corresponds to the right side of the image.

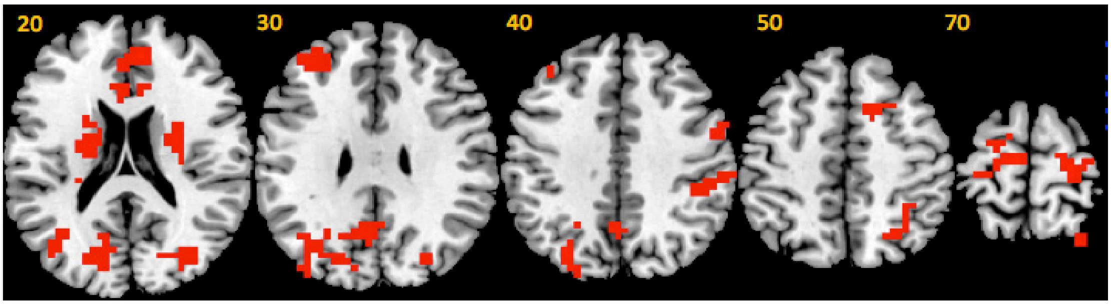

Supplementary Figure S5. Exploratory ANCOVA results for the between-group differences in brain activation for contrast comparing wins and losses at a liberal whole-brain cluster threshold (p<.05). Red indicates significant between-group differences in activation between adolescents with ADHD, adolescents with OCD, and healthy comparison adolescents. Talairach z-coordinates are indicated for slice distance (in mm) from the intercommissural line. The right side of the brain corresponds to the right side of the image.

Supplementary References

1. Carlisi C, Norman L, Murphy CM, Christakou A, Chantiluke K, Giampietro V, et al. (In Press): Shared and disorder-specific neurocomputational mechanisms of decision-making in Autism Spectrum Disorder and Obsessive-Compulsive Disorder. *Cereb Cortex*.
2. Christakou A, Brammer M, Giampietro V, Rubia K (2009): Right ventromedial and dorsolateral prefrontal cortices mediate adaptive decisions under ambiguity by integrating choice utility and outcome evaluation. *J Neurosci*. 29:11020-11028.
3. Christakou A, Gershman SJ, Niv Y, Simmons A, Brammer M, Rubia K (2013): Neural and psychological maturation of decision-making in adolescence and young adulthood. *J Cogn Neurosci*. 25:1807-1823.
4. Ahn WY, Busemeyer JR, Wagenmakers EJ, Stout JC (2008): Comparison of decision learning models using the generalization criterion method. *Cognitive science*. 32:1376-1402.
5. Tversky A, Kahneman D (1992): Advances in prospect theory: Cumulative representation of uncertainty. *Journal of Risk and Uncertainty*. 5:297-323.
6. Rescorla RA (1972): A theory of Pavlovian conditioning: The effectiveness of reinforcement and non-reinforcement. *Classical conditioning II: Current research and theory*.
7. Erev I, Roth AE (1998): Predicting How People Play Games: Reinforcement Learning in Experimental Games with Unique, Mixed Strategy Equilibria. *The American Economic Review*. 88:848-881.
8. Worthy DA, Hawthorne MJ, Otto AR (2013): Heterogeneity of strategy use in the Iowa gambling task: a comparison of win-stay/lose-shift and reinforcement learning models. *Psychonomic bulletin & review*. 20:364-371.
9. Carpenter B, Gelman A, Hoffman M, Lee D, Goodrich B, Betancourt M, et al. (2016): Stan: A probabilistic programming language. *J Stat Softw*.
10. Ahn W-Y, Haines N, Zhang L (2017): Revealing Neurocomputational Mechanisms of Reinforcement Learning and Decision-Making With the hBayesDM Package. *Computational Psychiatry*. 1:24-57.
11. Kruschke J (2014): *Doing Bayesian data analysis: A tutorial with R, JAGS, and Stan*. Academic Press.
12. Watanabe S (2010): Asymptotic equivalence of Bayes cross validation and widely applicable information criterion in singular learning theory. *Journal of Machine Learning Research*. 11:3571-3594.

13. Gelman A, Hwang J, Vehtari A (2014): Understanding predictive information criteria for Bayesian models. *Statistics and Computing*. 24:997-1016.
14. Carlisi CO, Chantiluke K, Norman L, Christakou A, Barrett N, Giampietro V, et al. (2016): The effects of acute fluoxetine administration on temporal discounting in youth with ADHD. *Psychological medicine*. 46:1197-1209.
15. Carlisi CO, Norman L, Murphy CM, Christakou A, Chantiluke K, Giampietro V, et al. (2017): Comparison of neural substrates of temporal discounting between youth with autism spectrum disorder and with obsessive-compulsive disorder. *Psychological medicine*. 47:2513-2527.
16. Carlisi CO, Norman L, Murphy CM, Christakou A, Chantiluke K, Giampietro V, et al. (In Press): Disorder-Specific and Shared Brain Abnormalities During Vigilance in Autism and Obsessive-Compulsive Disorder. *Biol Psychiatry Cogn Neurosci*.
17. Norman LJ, Carlisi CO, Christakou A, Chantiluke K, Murphy C, Simmons A, et al. (2017): Neural dysfunction during temporal discounting in paediatric Attention-Deficit/Hyperactivity Disorder and Obsessive-Compulsive Disorder. *Psychiatry Res Neuroimaging*. 269:97-105.
18. Norman LJ, Carlisi CO, Christakou A, Cubillo A, Murphy CM, Chantiluke K, et al. (2017): Shared and disorder-specific task-positive and default mode network dysfunctions during sustained attention in paediatric Attention-Deficit/Hyperactivity Disorder and obsessive/compulsive disorder. *NeuroImage : Clinical*. 15:181-193.
19. Rubia K, Halari R, Christakou A, Taylor E (2009): Impulsiveness as a timing disturbance: neurocognitive abnormalities in attention-deficit hyperactivity disorder during temporal processes and normalization with methylphenidate. *Philos Trans R Soc Lond B Biol Sci*. 364:1919-1931.
20. Murphy CM, Christakou A, Giampietro V, Brammer M, Daly EM, Ecker C, et al. (2017): Abnormal functional activation and maturation of ventromedial prefrontal cortex and cerebellum during temporal discounting in autism spectrum disorder. *Human Brain Mapping*. 38:5343-5355.
21. Chantiluke K, Christakou A, Murphy CM, Giampietro V, Daly EM, Ecker C, et al. (2014): Disorder-specific functional abnormalities during temporal discounting in youth with Attention Deficit Hyperactivity Disorder (ADHD), Autism and comorbid ADHD and Autism. *Psychiatry Research: Neuroimaging*. 223:113-120.
22. Murphy CM, Christakou A, Daly EM, Ecker C, Giampietro V, Brammer M, et al. (2014): Abnormal functional activation and maturation of fronto-striato-temporal and cerebellar regions during sustained attention in autism spectrum disorder. *Am J Psychiatry*. 171:1107-1116.

23. Christakou A, Murphy CM, Chantiluke K, Cubillo AI, Smith AB, Giampietro V, et al. (2013): Disorder-specific functional abnormalities during sustained attention in youth with Attention Deficit Hyperactivity Disorder (ADHD) and with Autism. *Molecular Psychiatry*. 18:236-244.

24. Brammer MJ, Bullmore ET, Simmons A, Williams SC, Grasby PM, Howard RJ, et al. (1997): Generic brain activation mapping in functional magnetic resonance imaging: a nonparametric approach. *Magnetic resonance imaging*. 15:763-770.

25. Bullmore E, Long C, Suckling J, Fadili J, Calvert G, Zelaya F, et al. (2001): Colored noise and computational inference in neurophysiological (fMRI) time series analysis: resampling methods in time and wavelet domains. *Hum Brain Mapp*. 12:61-78.

26. Bullmore ET, Suckling J, Overmeyer S, Rabe-Hesketh S, Taylor E, Brammer MJ (1999): Global, voxel, and cluster tests, by theory and permutation, for a difference between two groups of structural MR images of the brain. *IEEE Trans Med Imaging*. 18:32-42.

27. Thirion B, Pinel P, Meriaux S, Roche A, Dehaene S, Poline JB (2007): Analysis of a large fMRI cohort: Statistical and methodological issues for group analyses. *NeuroImage*. 35:105-120.

28. Figee M, Luigjes J, Smolders R, Valencia-Alfonso CE, van Wingen G, de Kwaasteniet B, et al. (2013): Deep brain stimulation restores frontostriatal network activity in obsessive-compulsive disorder. *Nat Neurosci*. 16:386-387.

29. Bullmore E, Long C, Suckling J, Fadili J, Calvert G, Zelaya F, et al. (2001): Colored noise and computational inference in neurophysiological (fMRI) time series analysis: resampling methods in time and wavelet domains. *Human Brain Mapping*. 12:61-78.

30. Talairach J, Tournoux P (1988): *Coplanar stereotaxic atlas of the human brain, a 3-dimensional proportional system: an approach to cerebral imaging*. New York: Thieme.
